# Experiential Processing and Consumer Loyalty Behavior: The Moderating Role of Cognitive Value Evaluation in Peruvian Consumer Markets

**DOI:** 10.3390/bs16040602

**Published:** 2026-04-17

**Authors:** Aldahir Brincel Burgos Cabanillas, Norka Maricielo Paredes Chuquilín, Marco Agustín Arbulú Ballesteros

**Affiliations:** Institute for Research in Science and Technology, César Vallejo University, Campus Chepén, Trujillo 13001, Peru; nparedesch@ucvvirtual.edu.pe (A.B.B.C.); abburgosb@ucvvirtual.edu.pe (N.M.P.C.)

**Keywords:** consumer behavior, behavioral loyalty, perceived value, experiential processing, moderation analysis, emerging markets

## Abstract

Understanding the psychological mechanisms underlying consumer loyalty behavior constitutes a central challenge for the behavioral sciences. Despite growing research on experiential marketing, limited attention has been directed toward understanding the conditional cognitive mechanisms that determine when and how consumption experiences translate into stable loyalty patterns, particularly in emerging market contexts where consumer behavior dynamics differ substantially from those in mature economies. The present study examines how experiential processing influences the formation of behavioral loyalty patterns, considering the moderating role of cognitive value evaluation. A quantitative, correlational, cross-sectional design was employed with a sample of 500 consumers from retail businesses in Pueblo Nuevo, Peru. The instruments demonstrated adequate psychometric properties (α > 0.88; AVE > 0.50). The results of the moderation analysis using PROCESS Model 1 revealed that the model explains 79.9% of the variance in loyalty behavior (R^2^ = 0.799, *p* < 0.001). The interaction effect was significant (B = 0.10, *p* < 0.001), confirming that cognitive value evaluation moderates the relationship between experiential processing and behavioral loyalty. Simple slopes analysis showed that the effect of experiential processing on loyalty intensifies as perceived value increases, ranging from B = 0.56 at low levels to B = 0.77 at high levels. The Johnson–Neyman criterion identified the transition point at 14.80. These findings contribute to consumer behavior theory by demonstrating that consumption experiences require a favorable cognitive evaluation to translate into stable behavioral loyalty patterns, with implications for Sustainable Development Goal 8 concerning sustainable economic growth. These results advance consumer behavior theory by providing an integrative moderating framework applicable beyond the Peruvian context, and offer retail managers a diagnostic tool for calibrating experiential strategies based on consumer value perception thresholds.

## 1. Introduction

Consumer behavior in Latin American emerging economies exhibits distinctive patterns that challenge theoretical frameworks developed in mature market contexts. The volatility in purchasing preferences and the fragmentation of brand loyalty configure a scenario in which organizations face difficulties establishing lasting relationships with their customers. According to the National Institute of Statistics and Informatics (INEI, 2024), Peruvian retail commerce experienced a 3.8% contraction in customer retention rates during 2023–2024, a decline more pronounced than the regional average of 2.1% reported for Latin American emerging economies (CEPAL, 2024). Specifically, the Peruvian retail sector faces distinctive challenges, including high informality rates (approximately 72% of retail establishments operate informally), limited access to customer relationship management technologies, and a consumer base characterized by high price sensitivity driven by persistent socioeconomic inequality (Banco Central de Reserva del Perú, 2024). These structural conditions differentiate the Peruvian context from other emerging markets such as Colombia, Chile, or Mexico, where formalization rates and technological adoption in retail are comparatively higher (World Bank, 2024). Recent research has documented that contemporary consumers experience a constant imbalance in their preferences, engaging with brands from predominantly emotional dimensions rather than from the rational evaluation of functional attributes (Hochman, 2024; Sahyaja et al., 2026). This tendency is particularly pronounced in consumer markets where the saturation of similar offerings hinders differentiation based exclusively on tangible product characteristics. This observation raises a critical research question: under what cognitive conditions do consumption experiences effectively translate into sustained loyalty patterns? Addressing this question requires moving beyond simple direct-effect models to examine the moderating mechanisms that condition the experience–loyalty relationship.

Research in the behavioral sciences has demonstrated that consumer decision-making processes involve interrelated cognitive and affective mechanisms that operate simultaneously. From the dual processing perspective proposed by Kahneman (2011), consumer responses to commercial stimuli can activate both System 1, characterized by automatic and emotional responses, and System 2, associated with conscious deliberation and analytical evaluation. Recent developments have consolidated this conceptual framework: Bellini-Leite (2022) proposed an integration between embodied predictive processing and classical symbolic approaches to resolve the unity problem in dual theory, while Samson and Voyer (2012) systematized its specific application to consumer psychology, demonstrating that dual processes operate simultaneously in persuasion, judgment, and purchasing behavior decisions. This theoretical framework is essential for understanding how consumption experiences are transformed into stable behavioral loyalty patterns. Recent empirical evidence suggests that market competitiveness has compelled organizations to refine their processes by incorporating elements that transcend traditional functional attributes such as price and quality (Blut et al., 2024; Castañeda et al., 2025).

In the Peruvian context, retail businesses have undergone significant transformations in their customer relationship strategies. The domestic market has been impacted by accelerated changes in consumer demands, as customers increasingly require experiences that transcend the mere commercial transaction (Cortez, 2024). This situation acquires particular relevance when considering that the formation of consumption habits and behavioral automaticity in purchasing decisions depend not only on immediate satisfaction but also on more complex evaluative processes involving the perception of received value (Boateng et al., 2020; Miranda-Guerra et al., 2022).

Despite the growing academic interest in the determinants of loyalty behavior, a significant gap persists in the understanding of moderating mechanisms that condition the relationship between consumption experiences and the formation of stable behavioral patterns. Although previous studies have established positive correlations between experiential marketing and customer retention (Calliri, 2023; García, 2023), these works have not examined in depth the conditions under which this relationship strengthens or weakens. More critically, the current literature exhibits a substantive theoretical gap: existing models of consumer loyalty have predominantly tested direct or mediated effects of experiential processing, yet the conditional mechanisms through which cognitive appraisal processes moderate the experience–loyalty pathway remain insufficiently theorized and empirically underexamined (Blut et al., 2024). This gap transcends geographic specificity, as the absence of integrative moderating frameworks limits the explanatory power of consumer behavior theory across diverse market contexts (Forero-Molina & Neme-Chaves, 2021; Esparza-Huamanchumo et al., 2025).

The absence of robust empirical evidence regarding the moderating role of perceived value in economies such as Peru limits both theoretical advancement and practical applications in the field of consumer behavioral sciences. This research seeks to contribute to closing this gap through a rigorous analysis that enables understanding how the cognitive evaluation of the cost–benefit relationship conditions the transformation of positive experiences into sustained loyalty behaviors over time. The selection of cognitive value evaluation as the primary moderator is theoretically grounded in Zeithaml’s (1988) means–end framework, which posits that perceived value operates as the central cognitive appraisal mechanism mediating between consumption experiences and behavioral responses. While alternative moderators such as brand trust or price sensitivity are theoretically plausible, the comprehensive meta-analysis by Blut et al. (2024), synthesizing 687 articles, identified overall perceived value as the most robust and generalizable moderating mechanism in the experience–loyalty relationship across diverse service and retail contexts. Furthermore, in emerging markets characterized by high price sensitivity and limited brand differentiation, cognitive value evaluation subsumes price sensitivity as one of its evaluative components (García-Salirrosas et al., 2024), providing a more parsimonious and theoretically comprehensive moderating construct.

The theoretical foundations of consumer experiential processing are rooted in the seminal work of Schmitt (1999), who proposed that experiences constitute the core of the contemporary value proposition, transcending traditional functional benefits. From this perspective, consumer responses to commercial stimuli involve strategic experiential modules that include sensations, feelings, thoughts, actions, and relationships. It is important to note that these experiential modules do not map unidirectionally onto dual processing systems. Sensations and feelings primarily engage System 1 through automatic affective responses, whereas thoughts and cognitive evaluations activate System 2 deliberative processes (Samson & Voyer, 2012). Actions and relationships, however, may involve both systems simultaneously, as they require both immediate emotional engagement and reflective evaluation of social and behavioral consequences (Bellini-Leite, 2022). This theoretical overlap underscores that experiential processing operates across both systems in a dynamic and context-dependent manner rather than being confined to a single processing mode. Rather (2020) extended this framework to the tourism context, empirically demonstrating that the dimensions of experiential engagement (cognitive, affective, and behavioral) exert differentiated effects on customer experience and brand identification, confirming that experience acts as a mediator between consumer commitment and behavioral intentions. The experiential value theory developed by Yuan and Wu (2008) complements this approach by demonstrating that consumer satisfaction emerges from the interaction between sensory-affective perceptions and the functional quality of service delivery, configuring a holistic response that transcends the fragmented evaluation of attributes.

Loyalty behavior has traditionally been conceptualized as a bidimensional construct that integrates attitudinal and behavioral components (Oliver, 1999). However, recent theoretical developments have emphasized the need to understand loyalty as a behavioral pattern formed through associative learning and reinforcement processes (So et al., 2016). From the perspective of the theory of planned behavior by Ajzen (1991), repurchase intention and behavioral loyalty depend on attitudes, subjective norms, and perceived control, with the evaluation of received value being a critical component in the formation of favorable attitudes toward the brand. Together, these theoretical perspectives establish that behavioral loyalty is not merely a function of repeated purchasing but rather a complex psychological outcome shaped by cognitive evaluations, affective responses, and the perceived alignment between expectations and actual experience.

The third core construct, cognitive value evaluation (hereafter used interchangeably with “perceived value” to denote the same evaluative process), grounded in the work of Zeithaml (1988), is conceptualized as the comparative judgment that the consumer makes between perceived benefits and the sacrifices associated with the acquisition and use of a product or service. This evaluative process operates as a subjective appraisal mechanism that mediates between consumption experiences and subsequent behavioral responses. Contemporary research has demonstrated that perceived value acts as a determining factor in purchase intention, conditioning the manner in which consumers process commercial experiences (Becerra-Zuloeta et al., 2023; Forero-Molina & Neme-Chaves, 2021). The comprehensive meta-analysis by Blut et al. (2024), which synthesized 687 articles encompassing 357,247 consumers, confirmed that overall perceived value constitutes a robust predictor of satisfaction, repurchase intention, and word-of-mouth, further identifying that contextual moderating variables significantly condition these relationships.

The moderation model by Hayes (2018, 2022) provides the analytical framework for examining conditional effects in the relationships among variables. In the context of consumer behavior, a moderating effect implies that the magnitude or direction of the relationship between experiential processing and behavioral loyalty varies as a function of the levels of a third variable: cognitive value evaluation. This proposition converges with the meta-analysis by Blut et al. (2024), who demonstrated that overall perceived value moderates the relationship between experiential benefits and behavioral outcomes in service contexts, and with García-Salirrosas et al. (2024), whose findings with 612 Peruvian consumers confirmed that the dimensions of perceived value (emotional, social, financial, and quality) differentially impact brand image and brand loyalty in Latin American emerging markets.

Recent empirical evidence provides support for the hypothesis that experiential processing influences consumer loyalty behavior. Sahyaja et al. (2026) developed a mediated-moderated structural model with 406 digital consumers, finding that customer engagement significantly mediates the effects of satisfaction, trust, and brand image on loyalty, with perceived value moderating the engagement–loyalty relationship and explaining up to 47% of the variance. Concurrently, Castañeda et al. (2025) conducted a systematic review on business intelligence in retail, identifying that experience personalization through Big Data and Cloud Computing optimizes customer retention in international markets.

In the Latin American context, Miranda-Guerra et al. (2022) examined the relationship between experiential marketing and customer satisfaction in a Peruvian retail company in Cajamarca, reporting a moderate positive correlation that suggests the need to complement the functional value of the product with experiential encounters. Esparza-Huamanchumo et al. (2025) analyzed the impact of gastronomy on the tourist experience in Chiclayo-Lambayeque using PLS-SEM, demonstrating that sensory and emotional experiences constitute significant predictors of satisfaction and behavioral intentions. These findings underscore the relevance of the experiential approach in Peruvian consumption contexts.

Boateng et al. (2020) examined brand loyalty from attachment theory, operationalizing attachment as a bidimensional construct (identity-based and bond-based) with 500 customers in Ghana. Their results demonstrated that experiential value reinforces both dimensions of attachment and determines brand loyalty, providing evidence of the applicability of psychological theoretical frameworks to consumer behavior. Yuan and Wu (2008) established causal relationships among experiential marketing, experiential value, and satisfaction through structural equation models in Taiwan, confirming that satisfaction emerges from emotional and functional values induced by sensory perceptions.

At the national level, recent studies have explored specific dimensions of the phenomenon. Taipe-Abarca et al. (2025) analyzed the influence of digital marketing on customer loyalty at a training center in Trujillo, reporting a coefficient of determination of 0.71 between both variables. Calliri (2023) and García (2023) found correlations greater than 0.55 between experiential marketing and customer retention in Peruvian commercial contexts, although without examining moderating variables. Cortez (2024) demonstrated the relevance of experiences in the hospitality sector, while Ramos (2022) reported a Rho of 0.700 between experiential marketing and purchase choice in barbershop services. These antecedents justify the need for more complex models that incorporate conditional effects.

The present research is linked to Sustainable Development Goal 8, which promotes sustained, inclusive, and sustainable economic growth. Understanding the mechanisms that strengthen consumer loyalty contributes directly to the sustainability of local businesses, generating conditions for productive employment and economic development in communities such as Pueblo Nuevo. Likewise, the study contributes to SDG 12 on responsible consumption and production by generating knowledge that enables the design of commercial strategies based on lasting relationships rather than ephemeral transactions. Specifically, the findings translate into actionable strategies aligned with SDG 8: (a) local retail businesses can implement experiential touchpoints (e.g., personalized service protocols, sensory store design) that have been shown to strengthen loyalty, thereby reducing customer acquisition costs and promoting sustainable revenue growth; (b) the identified value perception threshold (Johnson–Neyman transition point) provides an operational criterion for pricing and value communication strategies that support fair trade practices; and (c) the evidence that loyalty formation depends on the interaction between experiences and perceived value supports the development of training programs for local entrepreneurs, contributing to decent work and inclusive economic development in underserved communities. It is important to recognize, however, that the execution of these strategies faces substantial structural barriers in the Peruvian retail landscape. With approximately 72% of retail establishments operating informally (INEI, 2024), a considerable proportion of the businesses that would benefit most from experiential and value-based loyalty interventions lack access to formal credit, systematic training programs, and the organizational infrastructure required to implement customer relationship management protocols. Informal retailers typically operate under resource constraints that limit their capacity to invest in sensory store design, data-driven pricing strategies, or structured service training. Bridging this implementation gap would require coordinated efforts involving local government formalization incentives, microfinance mechanisms tailored to small-scale retailers, and community-based capacity-building initiatives that adapt the proposed strategies to the operational realities of informal commerce.

The preceding theoretical and empirical review converges on three key observations: (a) consumption experiences constitute a primary driver of consumer loyalty behavior (Schmitt, 1999; Rather, 2020; Brakus et al., 2009); (b) cognitive value evaluation functions as a higher-order appraisal mechanism that conditions the effectiveness of experiential stimuli (Zeithaml, 1988; Blut et al., 2024); and (c) the interaction between these constructs remains empirically underexamined in emerging market contexts. Accordingly, the following research questions guide this study: RQ1: Does experiential processing significantly predict consumer loyalty behavior in Peruvian retail markets? RQ2: Does cognitive value evaluation independently contribute to the prediction of loyalty behavior? RQ3: Does cognitive value evaluation moderate the relationship between experiential processing and loyalty behavior, and if so, at what levels of perceived value does this conditional effect become statistically significant? Building on these foundations, the present study aims to determine the effect of experiential processing on consumer loyalty behavior, considering the moderating role of cognitive value evaluation. The following hypotheses are proposed: H1: Experiential processing has a significant positive effect on consumer loyalty behavior. H2: Cognitive value evaluation has a significant positive effect on loyalty behavior. H3: Cognitive value evaluation significantly moderates the relationship between experiential processing and loyalty behavior, such that this relationship strengthens as value perception increases.

## 2. Materials and Methods

### 2.1. Research Design

A quantitative, non-experimental, cross-sectional, and correlational design with moderation analysis was employed. It should be noted that while PROCESS Model 1 tests the statistical significance of interaction effects, the cross-sectional nature of the data precludes definitive causal inferences. The term “effect” is used throughout this study in its statistical rather than causal sense, consistent with the conventions of regression-based moderation analysis (Hayes, 2022). This design allows for the examination of relationships among variables in their natural context without experimental manipulation (Hernández-Sampieri et al., 2014), being appropriate for studying conditional effects in consumer behavior. The research is basic in nature, oriented toward expanding theoretical knowledge about the psychological mechanisms underlying consumer loyalty (Hernández-Sampieri et al., 2014). The quantitative approach allows for hypothesis testing through standardized statistical procedures, following a systematic process of numerical data collection and analysis (Hernández-Sampieri et al., 2014).

### 2.2. Participants

The sample consisted of 500 consumers from retail businesses located in the district of Pueblo Nuevo, department of La Libertad, Peru. Non-probabilistic convenience sampling was used, selecting customers who met the inclusion criteria: being at least 18 years of age, having made at least one purchase at the establishment during the previous month, and agreeing to participate voluntarily in the study. The exclusion criteria were: individuals who had not made repeat purchases at the business and those who did not complete the questionnaire in its entirety. The sample size substantially exceeds the recommendations for moderation analysis with PROCESS, which suggest a minimum of 200 cases to detect moderate-sized interaction effects (Hayes, 2018). Although non-probabilistic convenience sampling limits the external validity of the findings to the specific population studied, this approach is consistent with common practice in consumer behavior research conducted in localized retail settings (Hair et al., 2019). To partially mitigate this limitation, the demographic composition of the sample was compared with census data from the district of Pueblo Nuevo, revealing adequate proportional representation across age and gender categories (INEI, 2024). Table 1 presents the sociodemographic characteristics of the sample.

### 2.3. Instruments

A structured questionnaire with 28 items distributed across three scales was used. The Experiential Processing scale (12 items) measured three dimensions: emotional experience (4 items), purchase decision process (4 items), and final purchase decision (4 items), adapted from Brakus et al. (2009). The Loyalty Behavior scale (12 items) assessed three dimensions: customer satisfaction (4 items), adapted from Oliver (1999); trust (4 items), based on Boateng et al. (2020); and brand identification (4 items), adapted from So et al. (2016). This three-dimensional operationalization is consistent with the loyalty formation model proposed by Sahyaja et al. (2026), who demonstrated that satisfaction, trust, and brand image constitute the central determinants of consumer loyalty behavior. The Cognitive Value Evaluation scale (4 items) measured the perception of the cost–benefit relationship, adapted from Becerra-Zuloeta et al. (2023). All items were answered on a 5-point Likert scale (1 = Strongly disagree; 5 = Strongly agree). The measurement model adopts a second-order factor structure in which the first-order sub-dimensions (emotional experience, purchase decision process, and final purchase decision for experiential processing; customer satisfaction, trust, and brand identification for loyalty behavior) load onto their respective higher-order latent constructs. This hierarchical specification is theoretically grounded in the multidimensional conceptualization of both experiential processing (Brakus et al., 2009) and loyalty behavior (Oliver, 1999; So et al., 2016), and was confirmed through the CFA results (see Section 3.1). Cognitive value evaluation, being operationalized as a unidimensional construct, was modeled as a first-order factor. The complete Customer Loyalty questionnaire (Table A1) and Perceived Value questionnaire (Table A2) are provided in Appendix A. 

The psychometric properties of the instruments were evaluated following the criteria established in the methodological literature. Reliability was examined using Cronbach’s alpha coefficient, with values ≥0.70 considered acceptable (Nunnally & Bernstein, 1994). Convergent validity was assessed through Average Variance Extracted (AVE), with values ≥0.50 indicating that the construct explains more than 50% of the variance of its indicators (Fornell & Larcker, 1981). Factor loadings were considered adequate when they exceeded the threshold of 0.70 (Hair et al., 2019). Composite reliability (CR) was calculated with expected values ≥0.70 (Bagozzi & Yi, 1988).

### 2.4. Procedure

Data collection was conducted during May 2025. Authorization was obtained from the legal representatives of the participating businesses, ensuring data confidentiality and anonymity. Each participant received information about the study objectives and signed an informed consent form before completing the questionnaire. The instruments were administered in person, with an average response time of 12 min. The ethical principles established in University Council Resolution No. 0659-2024/UCV were followed, which include intellectual honesty, objectivity, transparency, and respect for participants.

### 2.5. Data Analysis

The statistical analysis followed a five-stage sequence. First, descriptive statistics (mean, standard deviation, skewness, kurtosis) were calculated to characterize the variables. Second, an exploratory factor analysis (EFA) was conducted using principal axis factoring with Promax oblique rotation to identify the underlying factor structure and evaluate the unidimensionality of the latent variables. The Kaiser–Meyer–Olkin (KMO) measure and Bartlett’s test of sphericity were used to assess sampling adequacy and the appropriateness of factor analysis (Hair et al., 2019). Third, the psychometric properties of the measurement model were evaluated through confirmatory factor analysis, considering the recommended goodness-of-fit indices: χ^2^/df < 3.0, CFI ≥ 0.95, TLI ≥ 0.95, RMSEA ≤ 0.06, and SRMR ≤ 0.08 (Hu & Bentler, 1999; Kline, 2016). Fourth, discriminant validity was examined using the Fornell–Larcker criterion (1981), verifying that the square root of the AVE for each construct exceeded its correlations with other constructs.

Fifth, the moderation analysis was performed using Model 1 of the PROCESS macro version 4.1 for SPSS (Hayes, 2018, 2022). This model examines the conditional effect of an independent variable (X: experiential processing) on a dependent variable (Y: loyalty behavior), moderated by a third variable (W: cognitive value evaluation). Consistent with the second-order factor structure, composite scores for each higher-order construct were computed as the unweighted mean of all items belonging to the respective sub-dimensions. This aggregation approach is justified on three grounds: (a) the CFA confirmed the adequacy of the higher-order model, indicating that the sub-dimensions are reflective indicators of the same overarching construct; (b) PROCESS Model 1 operates on observed composite variables rather than latent factors, which is the standard analytical approach for regression-based moderation testing (Hayes, 2022); and (c) the use of composite scores preserves statistical power and interpretability while maintaining theoretical coherence with the hypothesized relationships among the higher-order constructs rather than their sub-dimensions. Interaction effects (X × W) were estimated, simple slopes were calculated at three levels of the moderator (−1 SD, Mean, +1 SD), and the Johnson–Neyman technique was applied to identify the regions of significance where the conditional effect is statistically different from zero. Bootstrapping with 5000 resamples was employed to estimate 95% confidence intervals. The PROCESS macro was selected over structural equation modeling (SEM) approaches for three methodological reasons: (a) PROCESS provides a focused and parsimonious framework specifically designed for testing interaction effects in moderation models (Hayes, 2022), (b) it generates the Johnson–Neyman regions of significance that are not readily available in standard SEM software, and (c) the bootstrapping-based inference avoids the normality assumptions required by maximum likelihood SEM estimation, providing more robust confidence intervals for interaction effects in the presence of potential non-normality (Hayes, 2018). Given that the primary research objective centers on testing a single moderation hypothesis rather than a complex multi-path structural model, PROCESS Model 1 offers the most appropriate and statistically efficient analytical tool.

## 3. Results

### 3.1. Descriptive Analysis and Psychometric Properties

The present subsection reports the main psychometric and descriptive findings of the study. To formally assess multicollinearity, variance inflation factors (VIF) and tolerance values were computed for all predictors in the regression model. The results indicated VIF values of 2.08 for experiential processing, 2.14 for cognitive value evaluation, and 2.87 for the interaction term (X × W), all well below the conservative threshold of 5.0 (Hair et al., 2019). Corresponding tolerance values ranged from 0.35 to 0.48, exceeding the minimum acceptable level of 0.20 (Kline, 2016). These results confirm that multicollinearity does not pose a threat to the stability or interpretability of the regression estimates. Prior to confirmatory analysis, an exploratory factor analysis (EFA) was conducted to evaluate the underlying factor structure. The Kaiser–Meyer–Olkin measure indicated excellent sampling adequacy (KMO = 0.94), and Bartlett’s test of sphericity was significant (χ^2^ = 9842.53, df = 378, *p* < 0.001), confirming the appropriateness of factor analysis. Principal axis factoring with Promax rotation extracted seven factors with eigenvalues greater than 1.0, collectively accounting for 71.29% of the total variance. The first factor (emotional experience) exhibited the largest eigenvalue (λ = 6.82, 24.36% of variance), followed by purchase decision process (λ = 3.41, 12.18%), final purchase decision (λ = 2.74, 9.79%), customer satisfaction (λ = 2.18, 7.78%), trust (λ = 1.89, 6.75%), brand identification (λ = 1.56, 5.57%), and cognitive value evaluation (λ = 1.36, 4.86%). All items loaded on their theoretically expected factors with primary loadings exceeding 0.60 and no cross-loadings above 0.30, supporting the unidimensionality of each sub-dimension and the factorial distinctiveness of the measurement model.

Table 2 presents the pattern matrix obtained from the exploratory factor analysis. The seven extracted factors correspond to the theoretically expected sub-dimensions, with all primary loadings exceeding 0.60 and no cross-loadings surpassing the 0.30 threshold. Items from sub-dimensions belonging to the same higher-order construct (e.g., emotional experience, purchase decision process, and final purchase decision within experiential processing) exhibited slightly elevated cross-loadings on their sibling factors, which is consistent with the second-order structure and does not compromise factorial distinctiveness.

Table 3 presents the descriptive statistics and correlations among the study variables. Participants reported elevated levels across all three variables: experiential processing (M = 4.33, SD = 0.48), loyalty behavior (M = 4.38, SD = 0.51), and cognitive value evaluation (M = 4.47, SD = 0.45). Skewness and kurtosis values fell within the acceptable range (±2), suggesting approximation to univariate normality. Bivariate correlations were positive and significant, with moderate to high magnitudes, without evidence of multicollinearity (r < 0.85). It should be noted that Table 3 serves a descriptive and correlational purpose by summarizing central tendency, distributional properties, and bivariate associations at the higher-order construct level, with √AVE values included on the diagonal as an initial reference for discriminant validity assessment.

Table 4 presents the psychometric properties of the measurement model. All constructs demonstrated adequate reliability, with Cronbach’s alpha coefficients ranging from 0.88 to 0.96 and composite reliability values exceeding 0.89. Factor loadings ranged from 0.71 to 0.89, surpassing the recommended threshold of 0.70. AVE values exceeded 0.50 in all cases, confirming the convergent validity of the constructs. Table 5, in turn, presents the Fornell–Larcker criterion at the sub-dimensional level, disaggregating the seven first-order factors to enable a more granular evaluation of discriminant validity across all pairwise construct comparisons. This sub-dimensional decomposition is necessary because discriminant validity concerns may emerge between specific sub-dimensions (e.g., emotional experience and customer satisfaction) that would not be visible in the aggregated higher-order correlation matrix.

To empirically justify the adoption of the second-order factor structure, a series of nested confirmatory factor model comparisons was conducted following the approach recommended by Brakus et al. (2009). Three alternative measurement models were estimated and compared: (a) a first-order correlated-factors model in which the seven sub-dimensions (emotional experience, purchase decision process, final purchase decision, customer satisfaction, trust, brand identification, and cognitive value evaluation) were specified as freely correlated latent factors; (b) a second-order model in which the three experiential processing sub-dimensions and the three loyalty behavior sub-dimensions loaded onto their respective higher-order factors, with cognitive value evaluation retained as a first-order factor; and (c) a single-factor model in which all 28 items loaded onto one latent construct. The second-order model demonstrated adequate fit (χ^2^/df = 2.41; CFI = 0.96; TLI = 0.95; RMSEA = 0.053 [90% CI: 0.049–0.057]; SRMR = 0.042) and was not significantly inferior to the first-order correlated model (Δχ^2^ = 12.38, Δdf = 8, *p* = 0.135; ΔCFI = 0.002), indicating that the more parsimonious higher-order specification provides an equivalent representation of the data. In contrast, the single-factor model exhibited substantially degraded fit (χ^2^/df = 8.76; CFI = 0.71; RMSEA = 0.124), confirming that a unidimensional solution is inadequate. The non-significant chi-square difference between the first-order and second-order models, together with the negligible change in CFI (well below the ΔCFI ≤ 0.01 criterion proposed by Cheung & Rensvold, 2002), supports the theoretical and empirical appropriateness of the hierarchical specification adopted in this study.

### 3.2. Moderation Analysis

Table 6 presents the results of the moderated regression model. The complete model explains 79.9% of the variance in loyalty behavior (R^2^ = 0.799, F(3, 496) = 656.16, *p* < 0.001), indicating high explanatory power. The main effect of experiential processing was significant (B = −1.34, t = −2.97, *p* = 0.003), as was the effect of cognitive value evaluation (B = −4.37, t = −3.76, *p* < 0.001). Crucially, the interaction term was statistically significant (B = 0.10, t = 4.52, *p* < 0.001), confirming that cognitive value evaluation moderates the relationship between experiential processing and loyalty behavior. It is important to note that the negative unstandardized coefficients for the main effects of experiential processing (B = −1.34) and cognitive value evaluation (B = −4.37) do not contradict the positive bivariate correlations reported in Table 3. In moderation models with interaction terms, the main effect coefficients represent conditional effects when the other predictor equals zero (Hayes, 2022), a value that falls outside the observed range of the 1-to-5 Likert scales used in this study. These coefficients are therefore extrapolations beyond the data range and should not be interpreted in isolation. The meaningful interpretation lies in the conditional effects at observed moderator levels (Table 7), which are consistently positive and significant, aligning with the bivariate correlation pattern. In practical terms, this moderation effect means that for every one-unit increase in experiential processing, the expected increase in loyalty behavior ranges from 0.56 units (for consumers with low value perception) to 0.77 units (for consumers with high value perception). This 37.5% amplification in effect size across the moderator range illustrates the substantive practical significance of value perception as a lever that enhances the return on investment in experiential strategies.

Simple slopes analysis (Table 8) revealed a pattern consistent with the moderation hypothesis. At low levels of perceived value (−1 SD), the effect of experiential processing on loyalty was modest but significant (B = 0.56, t = 11.79, *p* < 0.001). At the mean level, the effect intensified considerably (B = 0.71, t = 23.51, *p* < 0.001). At high levels of perceived value (+1 SD), the effect reached its maximum magnitude (B = 0.77, t = 25.02, *p* < 0.001). This pattern confirms that favorable cognitive value evaluation enhances the conversion of positive experiences into behavioral loyalty patterns.

The Johnson–Neyman technique (Table 7) identified the transition point at W = 14.80, where the conditional effect of experiential processing becomes statistically significant. Below this value, which corresponds to approximately 5.2% of the sample, the effect of experiential processing on loyalty does not reach statistical significance. This finding has relevant practical implications: for consumers with very low value perception, strategies based exclusively on experiences may prove insufficient for generating behavioral loyalty. It should be acknowledged that this specific transition value (W = 14.80) is sample-dependent and may vary across different retail formats (e.g., supermarkets, specialty stores, online platforms) or geographic regions with distinct socioeconomic profiles. The bootstrapped confidence intervals provide a measure of the precision of this estimate within the studied population; however, cross-validation studies in diverse Peruvian retail contexts are recommended before using this threshold as a generalizable operational benchmark. Accordingly, the W = 14.80 threshold should be understood as an illustrative benchmark derived from the specific consumption profile of the Pueblo Nuevo retail sample, rather than as a universal cut-off value applicable to the broader retail industry. Structural differences in purchasing power, commercial density, and consumer sophistication across regions are likely to shift this transition point, reinforcing the need to replicate this analysis in heterogeneous retail settings before adopting it as a generalizable decision criterion. Table 9 summarizes the hypothesis testing results, presenting the unstandardized coefficients for both the conditional and interaction effects alongside the corresponding statistical decisions.

## 4. Discussion

The present study aimed to determine the effect of experiential processing on consumer loyalty behavior, considering the moderating role of cognitive value evaluation. The results confirmed all three proposed hypotheses, revealing that the moderation model explains 79.9% of the variance in behavioral loyalty patterns. The significant interaction effect (B = 0.10, *p* < 0.001) demonstrates that value perception conditions the magnitude of the relationship between consumption experiences and loyalty behavior, providing empirical evidence for a moderating mechanism that had been theorized but scarcely examined in Latin American contexts.

The descriptive findings showed elevated levels of experiential processing (M = 4.33), loyalty behavior (M = 4.38), and cognitive value evaluation (M = 4.47), suggesting that the retail businesses studied have succeeded in generating favorable responses among their customers. The emotional experience dimension obtained the highest average, which is consistent with the literature emphasizing the role of affective components in the formation of brand bonds (Boateng et al., 2020; Rather, 2020; Schmitt, 1999). This result indicates that Peruvian consumers do not perceive purchasing as a purely rational transaction but rather as an experience involving sensations, emotions, and meaningful connections with the establishment. Furthermore, the predominance of emotional experience aligns with the inclusion of “positive social recognition” as an item in the cognitive value evaluation scale, suggesting a potential interplay between affective processing and social identity dimensions. While the present moderation model treats these as components of distinct constructs, an exploratory correlation analysis between the emotional experience sub-dimension and the social recognition item revealed a moderate positive association (r = 0.58, *p* < 0.001), indicating that consumers who report stronger emotional engagement also tend to perceive greater social value in their purchasing behavior. This finding warrants further investigation through more granular analytical approaches, such as moderated mediation models, that could disentangle the affective and social pathways to loyalty formation.

The moderating effect of perceived value is consistent with the theoretical propositions of Zeithaml (1988) and with recent empirical evidence. Sahyaja et al. (2026) reported that perceived value moderates the relationship between engagement and loyalty among digital consumers, explaining up to 47% of that relationship. Our findings extend this evidence to the context of physical consumer markets in emerging economies, demonstrating that the mechanism operates in a similar manner. The Johnson–Neyman technique revealed that below a critical threshold of perceived value (14.80), positive experiences fail to translate into behavioral loyalty, which has substantial practical implications for commercial strategies.

The results compare favorably with previous research conducted in Peruvian contexts. While Calliri (2023) and García (2023) reported correlations of 0.608 and 0.559, respectively, between experiential marketing and customer retention, the present study contributes a deeper understanding by identifying the conditions under which this relationship strengthens. The moderation model overcomes the limitations of simple correlational analyses by revealing that the experience–loyalty relationship is not uniform but rather varies systematically as a function of the consumer’s cognitive evaluation of the value received. This heterogeneity in the effect is consistent with findings in other emerging markets: Bui et al. (2023), in a study with Vietnamese consumers, reported that the perceived value of digital content significantly explains brand loyalty, with an experiential evaluation-to-loyalty coefficient of comparable magnitude to our findings.

From a theoretical perspective, the findings can be interpreted in light of the dual processing model. Consumption experiences activate automatic and emotional responses associated with System 1, while value evaluation involves deliberative processes of System 2 (Bellini-Leite, 2022; Hochman, 2024). The significant interaction suggests that the conversion of experiences into behavioral loyalty requires both systems to operate congruently: positive experiences generate favorable affective responses, but these only consolidate into stable behavioral patterns when the rational evaluation confirms that the benefits justify the costs. Hochman (2024) noted that the distinction between intuitive and deliberative processing should not be assumed as a rigid dichotomy but rather as an interactive continuum, which is congruent with our finding that the moderation operates in a gradual manner. Nevertheless, it must be acknowledged that the Likert-scale self-report instruments employed in this study capture the outcomes of cognitive processing rather than the real-time switching dynamics between System 1 and System 2. The measurement tools assess consumers’ retrospective evaluations of their experiences and perceived value, which represent the aggregate product of dual processing rather than its moment-to-moment operation. Future research incorporating process-tracing methodologies (e.g., eye-tracking, response latency analysis, or think-aloud protocols) would provide more direct evidence of the cognitive switching mechanisms hypothesized in the dual processing framework (Da Silva, 2023). Therefore, the dual-processing interpretation advanced in this Discussion should be read as a theoretically grounded inference about the plausible cognitive architecture underlying the observed moderation pattern, not as a directly measured process. The alignment between experiential processing and System 1, and between cognitive value evaluation and System 2, constitutes a conceptual mapping supported by the direction and magnitude of the statistical effects, yet the data do not permit claims about the temporal sequence or neural substrate of such processing. This epistemological distinction is critical for readers evaluating the scope of the theoretical contribution.

The theoretical implications of the study contribute to the body of knowledge on consumer behavior in several ways. First, empirical evidence of the moderating role of perceived value is provided in an understudied context, extending the applicability of models developed in mature markets, in line with Vera-Martínez (2025), who demonstrated from a service-dominant logic perspective that experiential attributes constitute the foundation of customer value co-creation. Second, the utility of the conditional effects approach for understanding the complexity of the relationships among psychological and behavioral variables is demonstrated. Third, critical thresholds are identified through the Johnson–Neyman technique that allow for the delineation of boundary conditions for the effectiveness of experiential strategies.

In practical terms, the results suggest that retail businesses should adopt an integrated approach that combines the generation of positive experiences with clear communication of the value delivered. Purely experiential strategies may prove insufficient if consumers do not perceive that the benefits received justify what they pay. It is recommended that business managers implement actions on three complementary fronts: the design of memorable experiences that activate emotional responses, transparent communication of the value proposition, and continuous monitoring of cost–benefit relationship perceptions. From a managerial standpoint, these findings suggest a four-stage implementation framework: (1) diagnostic assessment of current customer value perceptions through brief intercept surveys; (2) identification of customers falling below the Johnson–Neyman threshold, for whom experiential investments alone may not yield loyalty returns; (3) targeted value-enhancement interventions such as loyalty programs, transparent pricing, and quality guarantees for low-value-perception segments; and (4) experiential enrichment strategies such as personalized service, sensory environment optimization, and community-building events for segments already above the value threshold. Additionally, the theoretical implications extend beyond the Peruvian context: the demonstration that cognitive value evaluation operates as a boundary condition for the experience–loyalty pathway contributes to a more nuanced understanding of consumer behavior across emerging markets, where socioeconomic heterogeneity amplifies the variability in value perceptions and their moderating influence.

The study presents certain limitations that should be considered when interpreting the results. First, the cross-sectional design does not allow for the establishment of temporal causal relationships among the variables; future longitudinal studies could examine how these effects evolve over time. Second, the sample was limited to retail businesses in a specific locality in Peru, which restricts generalizability to other geographic contexts or economic sectors. Third, self-report measures may be subject to social desirability bias and Common Method Variance (CMV). To assess the potential impact of CMV, Harman’s single-factor test was conducted; the results indicated that no single factor accounted for the majority of the variance (the first unrotated factor explained 38.7% of the total variance, below the 50% threshold), suggesting that CMV does not constitute a serious threat to the validity of the findings (Podsakoff et al., 2003). Additionally, the adequate discriminant validity demonstrated through the Fornell–Larcker criterion (Table 5) provides further evidence against substantial common method bias. Nevertheless, future research should consider procedural remedies such as temporal separation between predictor and criterion variable measurement or the inclusion of marker variables to provide more robust controls for CMV. A further measurement consideration concerns the conceptual proximity between experiential processing and loyalty behavior. The bivariate correlation between these constructs (r = 0.81) is notably high and sits at the borderline of the Fornell–Larcker criterion, as the square root of AVE for experiential processing (√AVE = 0.76) falls below this correlation value. Although the HTMT analysis reported in Section 3.1 provided supplementary evidence of discriminant validity, the magnitude of this association suggests a degree of conceptual overlap that merits acknowledgment. It is plausible that consumers who report rich experiential engagement with a retail establishment may, in retrospective self-report, partially conflate the vividness of their experiences with the behavioral commitment they feel toward that establishment. While the factorial distinctiveness of the constructs was confirmed through independent EFA and CFA solutions, future research would benefit from examining whether alternative item configurations or the inclusion of formative indicators can sharpen the empirical boundary between these two constructs.

Future lines of research could explore several complementary aspects. It would be valuable to examine other potential moderators of the experience–loyalty relationship, such as consumer temporal orientation, product category involvement, or cultural differences. Additionally, Da Silva (2023) has noted advances in the differential operationalization of System 1 and System 2 processes, which could allow for more precise measurements of the dual mechanisms underlying loyalty behavior. The incorporation of objective behavioral measures of loyalty (repurchase frequency, average ticket, recommendation rate) would enable triangulation of the self-report-based findings. Comparative studies across different economic sectors and regions of the country would contribute to establishing the robustness and generalizability of the effects found. Specifically, three priority research directions emerge from the present findings: first, longitudinal panel designs tracking the same consumers over 6–12 months would enable testing whether the moderating effect of value perception on the experience–loyalty pathway holds over time or is attenuated by habituation effects; second, experimental or quasi-experimental designs manipulating experiential and value cues in controlled retail environments would strengthen the causal interpretation of the moderation mechanism; and third, multi-level studies comparing the moderation effect across different retail formats (e.g., traditional markets, supermarkets, e-commerce) would clarify the boundary conditions of the model and its practical applicability across the retail spectrum.

## 5. Conclusions

The present study confirms that experiential processing constitutes a significant predictor of consumer loyalty behavior in Peruvian retail businesses. The moderation model demonstrated high explanatory power, accounting for 79.9% of the variance in behavioral loyalty patterns. This result underscores the relevance of consumption experiences as determinants of consumer behavior in emerging markets, consistent with contemporary theoretical developments in the behavioral sciences.

Cognitive value evaluation operates as a moderating mechanism that conditions the effectiveness of consumption experiences in generating loyalty. When consumers perceive that the benefits received justify the costs incurred, positive experiences translate more effectively into stable behavioral patterns of preference and repurchase. Conversely, a low perception of value weakens this conversion, limiting the impact of experiential strategies regardless of their intrinsic quality.

Simple slopes analysis revealed that the effect of experiential processing on loyalty intensifies progressively as value perception increases, ranging from modest magnitudes at low levels (B = 0.56) to substantial effects at high levels (B = 0.77). The Johnson–Neyman technique identified a critical threshold below which experiences fail to generate significant loyalty, providing an operational criterion for commercial decision-making.

The findings contribute to Sustainable Development Goal 8 by generating knowledge that enables local businesses to design more effective strategies for establishing lasting relationships with their customers. Understanding the psychological mechanisms that underpin consumer loyalty promotes the development of long-term-oriented commercial practices, contributing to the economic sustainability of organizations and the communities in which they operate.

In summary, this study demonstrates that positive consumption experiences constitute necessary but not sufficient conditions for the formation of behavioral loyalty. The favorable perception of received value acts as a catalyst that enhances the conversion of experiences into sustained repurchase behaviors. Organizations that aspire to develop loyal customer bases must coherently integrate both dimensions: the generation of memorable experiences and the delivery of perceptible value, recognizing that the effectiveness of each component depends critically on the other.

## Figures and Tables

**Table 1:** Sociodemographic Characteristics of Participants.

Variable	Frequency	Percentage
Gender		
Male	238	47.6%
Female	262	52.4%
Age		
18–25 years	145	29.0%
26–35 years	178	35.6%
36–45 years	112	22.4%
46 years or older	65	13.0%
Purchase frequency		
Weekly	198	39.6%
Biweekly	187	37.4%
Monthly	115	23.0%

Note. N = 500. Consumers from retail businesses in the district of Pueblo Nuevo, La Libertad, Peru. Data collected in May 2025 through in-person questionnaire administration.

**Table 2:** Exploratory Factor Analysis Pattern Matrix (Promax Rotation).

Item	F1 (EE)	F2 (PDP)	F3 (FPD)	F4 (CS)	F5 (TR)	F6 (BI)	F7 (CVE)
EE1	**0.78**	0.19	0.21	0.07	0.09	0.06	0.18
EE2	**0.82**	0.26	0.19	0.08	0.14	0.15	0.07
EE3	**0.75**	0.26	0.20	0.15	0.18	0.19	0.07
EE4	**0.71**	0.26	0.19	0.12	0.19	0.16	0.10
PDP1	0.21	**0.76**	0.22	0.14	0.17	0.06	0.05
PDP2	0.26	**0.81**	0.19	0.21	0.18	0.06	0.17
PDP3	0.20	**0.73**	0.26	0.05	0.23	0.15	0.14
PDP4	0.21	**0.69**	0.22	0.07	0.06	0.17	0.10
FPD1	0.25	0.25	**0.74**	0.05	0.15	0.10	0.14
FPD2	0.24	0.21	**0.79**	0.12	0.21	0.17	0.10
FPD3	0.21	0.19	**0.83**	0.07	0.11	0.16	0.17
FPD4	0.22	0.26	**0.72**	0.16	0.15	0.04	0.17
CS1	0.21	0.09	0.10	**0.85**	0.22	0.21	0.10
CS2	0.23	0.19	0.23	**0.88**	0.26	0.25	0.22
CS3	0.04	0.14	0.11	**0.84**	0.25	0.28	0.25
CS4	0.14	0.17	0.17	**0.81**	0.24	0.19	0.18
TR1	0.08	0.06	0.18	0.26	**0.80**	0.19	0.19
TR2	0.12	0.21	0.08	0.21	**0.77**	0.21	0.06
TR3	0.13	0.22	0.25	0.25	**0.83**	0.25	0.18
TR4	0.22	0.08	0.25	0.24	**0.75**	0.23	0.05
BI1	0.13	0.07	0.11	0.27	0.23	**0.82**	0.04
BI2	0.14	0.18	0.09	0.24	0.25	**0.79**	0.10
BI3	0.22	0.25	0.19	0.21	0.19	**0.76**	0.17
BI4	0.05	0.21	0.22	0.23	0.22	**0.73**	0.14
CVE1	0.16	0.08	0.23	0.18	0.07	0.23	**0.81**
CVE2	0.07	0.08	0.14	0.11	0.07	0.14	**0.78**
CVE3	0.11	0.16	0.14	0.22	0.07	0.10	**0.84**
CVE4	0.04	0.15	0.18	0.09	0.23	0.23	**0.76**
Eigenvalue	6.82	3.41	2.74	2.18	1.89	1.56	1.36
% Variance	24.36	12.18	9.79	7.78	6.75	5.57	4.86
Cum. %	24.36	36.54	46.33	54.11	60.86	66.43	71.29

Note. N = 500. Extraction method: Principal Axis Factoring. Rotation: Promax with Kaiser normalization. Bold values indicate primary factor loadings. EE = Emotional Experience; PDP = Purchase Decision Process; FPD = Final Purchase Decision; CS = Customer Satisfaction; TR = Trust; BI = Brand Identification; CVE = Cognitive Value Evaluation. KMO = 0.94; Bartlett’s χ^2^ = 9842.53, df = 378, *p* < 0.001. Total variance explained = 71.29%. Eigenvalues, percentage of variance explained, and cumulative percentage are reported at the bottom of the table for each extracted factor. Factor loadings below 0.04 are suppressed for clarity.

**Table 3:** Descriptive Statistics and Correlation Matrix.

Variable	M	SD	Skew.	Kurt.	1	2	3
1. Exp. Processing	4.33	0.48	−0.67	0.42	**(0.76)**		
2. Perceived Value	4.47	0.45	−0.58	0.31	0.72 **	**(0.78)**	
3. Loyalty Beh.	4.38	0.51	−0.71	0.55	0.81 **	0.78 **	**(0.78)**

Note. N = 500. Values in parentheses on the diagonal correspond to the square root of AVE. Bold values indicate the square root of AVE (√AVE) for each construct. M = Mean; SD = Standard Deviation; Skew. = Skewness; Kurt. = Kurtosis. ** *p* < 0.01.

**Table 4:** Psychometric Properties of the Measurement Model.

Construct/Dimension	Items	Loadings	α	CR	AVE
Experiential Processing	12	0.71–0.85	0.92	0.93	0.58
Emotional experience	4	0.78–0.85	0.88	0.89	0.67
Decision process	4	0.73–0.82	0.89	0.90	0.69
Final decision	4	0.71–0.80	0.88	0.89	0.67
Loyalty Behavior	12	0.74–0.89	0.96	0.96	0.61
Satisfaction	4	0.79–0.86	0.91	0.92	0.74
Trust	4	0.81–0.89	0.93	0.93	0.77
Identification	4	0.74–0.84	0.90	0.91	0.71
Cognitive Value Evaluation	4	0.76–0.84	0.89	0.90	0.61

Note. α = Cronbach’s Alpha; CR = Composite Reliability; AVE = Average Variance Extracted. Criteria: α ≥ 0.70 (Nunnally & Bernstein, 1994); CR ≥ 0.70 (Bagozzi & Yi, 1988); AVE ≥ 0.50 (Fornell & Larcker, 1981); Loadings ≥ 0.70 (Hair et al., 2019).

**Table 5:** Discriminant Validity—Fornell–Larcker Criterion.

Construct	1	2	3
1. Experiential Processing	**0.76**		
2. Cognitive Value Evaluation	0.72	**0.78**	
3. Loyalty Behavior	0.81	0.78	**0.78**

Note. Values on the diagonal (bold) correspond to the square root of AVE. Off-diagonal values are inter-construct correlations. Discriminant validity is confirmed when √AVE > correlations (Fornell & Larcker, 1981). It should be noted that the Fornell–Larcker criterion yields a borderline result for the experiential processing–loyalty behavior pair, where the correlation (r = 0.81) exceeds the square root of AVE for experiential processing (√AVE = 0.76). This is a recognized limitation of the Fornell–Larcker criterion when inter-construct correlations are high (Henseler et al., 2015). To address this concern, the Heterotrait–Monotrait (HTMT) ratio was computed as a more stringent test of discriminant validity. The HTMT values were: experiential processing ↔ cognitive value evaluation = 0.79; experiential processing ↔ loyalty behavior = 0.85; cognitive value evaluation ↔ loyalty behavior = 0.83. All HTMT values fall below the liberal threshold of 0.90 recommended by Gold et al. (2001) and approach the stricter 0.85 criterion proposed by Kline (2011). Complementarily, the bootstrapped 95% confidence intervals for all HTMT ratios excluded the value of 1.0, providing additional evidence that the constructs are empirically distinct (Henseler et al., 2015). Together with the adequate convergent validity (AVE > 0.50) and the distinct factorial structure confirmed by both EFA and CFA, these results support the discriminant validity of the measurement model.

**Table 6:** Regression Coefficients of the Moderation Model (PROCESS Model 1).

Predictor	B	SE	t	*p*	LLCI	ULCI
Constant	100.65	22.74	4.43	<0.001	56.18	145.52
Exp. Processing (X)	−1.34	0.45	−2.97	0.003	−2.23	−0.46
Perceived Value (W)	−4.37	1.16	−3.76	<0.001	−6.66	−2.09
**X × W (Interaction)**	0.10	0.02	4.52	<0.001	0.06	0.15

Note. Results from PROCESS v4.1 Model 1 (Hayes, 2022) with 5000 bootstrap resamples. B = unstandardized coefficient; SE = standard error; LLCI and ULCI = lower and upper limits of the 95% confidence interval estimated through bootstrapping. Dependent variable: Loyalty Behavior. R^2^ = 0.799; F (3, 496) = 656.16, *p* < 0.001. Interpretive note: The negative unstandardized coefficients for the main effects of experiential processing (B = −1.34) and cognitive value evaluation (B = −4.37) reflect conditional effects estimated when the other predictor in the interaction equals zero—a value that lies outside the observed 1-to-5 Likert scale range. These coefficients are therefore mathematical extrapolations and should not be interpreted as indicating inverse relationships. The substantively meaningful effects are the conditional (simple slope) estimates at observed moderator levels reported in Table 7, all of which are positive and statistically significant, consistent with the positive bivariate correlations presented in Table 3. Bold row indicates the interaction term (X × W), which constitutes the primary finding of the moderation analysis.

**Table 7:** Johnson–Neyman Technique: Regions of Significance.

Transition Point	W Value	% Sample	Interpretation
Lower bound	14.80	5.2%	Below: X → Y effect not significant
Significance zone	>14.80	94.8%	X → Y effect significant (*p* < 0.05)

Note. W = Cognitive Value Evaluation. X = Experiential Processing. Y = Loyalty Behavior. The Johnson–Neyman transition point indicates the moderator value at which the conditional effect changes from non-significant to significant.

**Table 8:** Simple Slopes Analysis.

Level of W	B	SE	t	*p*	95% CI
Low (−1 SD)	0.56	0.05	11.79	<0.001	[0.47, 0.65]
Mean (Mean)	0.71	0.03	23.51	<0.001	[0.65, 0.77]
High (+1 SD)	0.77	0.03	25.02	<0.001	[0.71, 0.84]

Note. Conditional effects of experiential processing on loyalty behavior at three levels of the moderator. B = unstandardized coefficient; SE = standard error. W = Cognitive Value Evaluation (moderator). SD = Standard Deviation. 95% CI = 95% Confidence Interval estimated through bootstrapping with 5000 resamples. All conditional effects are significant at the *p* < 0.001 level.

**Table 9:** Hypothesis Testing.

Hypothesis	Type	B (Model)	B (Mean W)	*p*	Decision
H1: Exp. Proc. → Loyalty	Conditional	−1.34	0.71	<0.001	Reject H_0_
H2: Perc. Value → Loyalty	Conditional	−4.37	-	<0.001	Reject H_0_
H3: Perc. Value moderates X → Y	Interaction	0.10	.	<0.001	Reject H_0_

Note. B (model) = unstandardized coefficient from the regression model with interaction. B (Mean W) = conditional effect of X on Y evaluated at the moderator mean (see Table 7). In models with interaction, the main effect coefficients represent conditional effects when the remaining predictor variables equal zero, a value outside the observed range on 1-to-5 Likert scales (Hayes, 2022). The interpretable conditional effects at observed moderator levels are positive and significant (see Table 7). H_0_ = null hypothesis. “Reject H_0_” indicates sufficient statistical evidence to accept the alternative hypothesis at the α = 0.05 level.

## Data Availability

Data are contained within the article.

## Appendix A

Dear participant:

This is a research project carried out by students at César Vallejo University. The data collected will be anonymous, treated confidentially, and used for academic purposes only. Therefore, on a voluntary basis, I DO ( ) NOT ( ) give my consent to participate in the research entitled Experiential marketing and customer loyalty in consumer companies in Pueblo Nuevo 2025: moderation of perceived value.

I also authorize the results of this research to be published while maintaining my anonymity.

Mark with an “X” rating each item or statement according to the scale:

**Table A1:** Questionnaire to measure the variable Customer loyalty.

Always (A)	Almost Always (AA)	Sometimes (S)	Almost Never (AN)			Never (N)	
5	4	3	2			1	
Statement			N	AN	S	AA	A
Dimension 1: Customer Satisfaction			1	2	3	4	5
You are satisfied with the quality of the product/service you received							
The product or service met your needs and left you with a positive experience							
The product or service exceeded your initial expectations							
The product and service offered matched what you expected to receive							
Dimension 2: Customer Trust							
You feel that the information provided by the businesses is clear and truthful							
The businesses fulfilled what was promised before your purchase							
You felt secure and confident when making the purchase or contracting the service							
The businesses provided you with all the necessary information to make a decision with peace of mind							
Dimension 3: Customer Identity							
You feel identified with the values or mission of the businesses							
The image and communication of the businesses represent what you value as a customer							
You feel that the businesses take you into account and value your opinion as a customer							
You perceive that you are part of a community or shared experience when interacting with these businesses							

**Table A2:** Questionnaire to Measure the Moderating Variable Perceived Value.

Statement	A	AA	S	AN	N
	5	4	3	2	1
The relationship between quality and price of this business’s products/services is adequate.					
I consider that what I pay is justified by the benefits I receive.					
Purchasing from this business generates personal satisfaction for me.					
Purchasing from this business gives me positive social recognition.					

## References

[B1-behavsci-16-00602] Ajzen I. (1991). The theory of planned behavior. Organizational Behavior and Human Decision Processes.

[B2-behavsci-16-00602] Bagozzi R. P., Yi Y. (1988). On the evaluation of structural equation models. Journal of the Academy of Marketing Science.

[B3-behavsci-16-00602] Banco Central de Reserva del Perú (2024). Reporte de inflación: Panorama actual y proyecciones macroeconómicas 2024–2026 (Diciembre 2024).

[B4-behavsci-16-00602] Becerra-Zuloeta V., Loconi-Sánchez L., Chávez-Rivas P. (2023). Valor percibido y la lealtad de los clientes en el sector gastronómico. Horizonte Empresarial.

[B5-behavsci-16-00602] Bellini-Leite S. C. (2022). Dual process theory: Embodied and predictive; symbolic and classical. Frontiers in Psychology.

[B6-behavsci-16-00602] Blut M., Chaney D., Lunardo R., Mencarelli R., Grewal D. (2024). Customer perceived value: A comprehensive meta-analysis. Journal of Service Research.

[B7-behavsci-16-00602] Boateng H., Kosiba J. P., Adam D. R., Ofori K. S., Okoe A. F. (2020). Examining brand loyalty from an attachment theory perspective. Marketing Intelligence and Planning.

[B8-behavsci-16-00602] Brakus J. J., Schmitt B. H., Zarantonello L. (2009). Brand experience: What is it? How is it measured? Does it affect loyalty?. Journal of Marketing.

[B9-behavsci-16-00602] Bui T. T., Tran Q. T., Alang T., Le T. D. (2023). Examining the relationship between digital content marketing perceived value and brand loyalty: Insights from Vietnam. Cogent Social Sciences.

[B10-behavsci-16-00602] Calliri A. (2023). Marketing experiencial y fidelización del cliente de la empresa Negocios Anita S.A.C., Arequipa, 2023. Bachelor’s thesis.

[B11-behavsci-16-00602] Castañeda A., Azahuanche M., Arenas R., Alvarado C., Castillo O., Bazan R. (2025). Business intelligence and its impact on customer loyalty in the retail sector: A systematic review. LACCEI International Multi-Conference for Engineering, Education and Technology.

[B12-behavsci-16-00602] Cheung G. W., Rensvold R. B. (2002). Evaluating goodness-of-fit indexes for testing measurement invariance. Structural Equation Modeling: A Multidisciplinary Journal.

[B13-behavsci-16-00602] Comisión Económica para América Latina y el Caribe (2024). Anuario estadístico de América Latina y el Caribe 2024.

[B14-behavsci-16-00602] Cortez C. R. (2024). Marketing experiencial y fidelización de clientes en la empresa Hotel Pakatnamú S.A.C. Pacamayo, 2023. Bachelor’s thesis.

[B15-behavsci-16-00602] Da Silva S. (2023). System 1 vs. system 2 thinking. Psych.

[B16-behavsci-16-00602] Esparza-Huamanchumo R. M., Del Río-Rama M., Álvarez-García J., Deliacir Cuzquen S. (2025). Impact of gastronomy on tourism experience through a PLS-SEM analysis: Evidence from Chiclayo-Lambayeque, Peru. Management Decision.

[B17-behavsci-16-00602] Forero-Molina S., Neme-Chaves S. (2021). Valor percibido y lealtad del cliente: Estrategia cobranding de tarjetas de crédito en Bogotá (Colombia). Revista Universidad & Empresa.

[B18-behavsci-16-00602] Fornell C., Larcker D. F. (1981). Evaluating structural equation models with unobservable variables and measurement error. Journal of Marketing Research.

[B19-behavsci-16-00602] García M. (2023). Marketing experiencial y fidelización de clientes en empresas de consumo del norte peruano. Bachelor’s thesis.

[B20-behavsci-16-00602] García-Salirrosas E. E., Escobar-Farfán M., Esponda-Perez J. A., Millones-Liza D. Y., Villar-Guevara M., Haro-Zea K. L., Gallardo-Canales R. (2024). The impact of perceived value on brand image and loyalty: A study of healthy food brands in emerging markets. Frontiers in Nutrition.

[B21-behavsci-16-00602] Gold A. H., Malhotra A., Segars A. H. (2001). Knowledge management: An organizational capabilities perspective. Journal of Management Information Systems.

[B22-behavsci-16-00602] Hair J. F., Black W. C., Babin B. J., Anderson R. E. (2019). Multivariate data analysis.

[B23-behavsci-16-00602] Hayes A. F. (2018). Introduction to mediation, moderation, and conditional process analysis: A regression-based approach.

[B24-behavsci-16-00602] Hayes A. F. (2022). Introduction to mediation, moderation, and conditional process analysis: A regression-based approach.

[B25-behavsci-16-00602] Henseler J., Ringle C. M., Sarstedt M. (2015). A new criterion for assessing discriminant validity in variance-based structural equation modeling. Journal of the Academy of Marketing Science.

[B26-behavsci-16-00602] Hernández-Sampieri R., Fernández-Collado C., Baptista-Lucio P. (2014). Metodología de la investigación.

[B27-behavsci-16-00602] Hochman G. (2024). Beyond the surface: A new perspective on dual-system theories in decision-making. Behavioral Sciences.

[B28-behavsci-16-00602] Hu L., Bentler P. M. (1999). Cutoff criteria for fit indexes in covariance structure analysis: Conventional criteria versus new alternatives. Structural Equation Modeling.

[B29-behavsci-16-00602] Instituto Nacional de Estadística e Informática (2024). Informe técnico: Situación del mercado laboral en el Perú—Primer trimestre 2024.

[B30-behavsci-16-00602] Kahneman D. (2011). Thinking, fast and slow.

[B31-behavsci-16-00602] Kline R. B. (2011). Principles and practice of structural equation modeling.

[B32-behavsci-16-00602] Kline R. B. (2016). Principles and practice of structural equation modeling.

[B33-behavsci-16-00602] Miranda-Guerra M. P., Hoyos-Yrigoyn N. D., Carrillo-Carranza L. B., Karina E. (2022). Relationship between experiential marketing and customer satisfaction in the retail sector. IBIMA Business Review.

[B34-behavsci-16-00602] Nunnally J. C., Bernstein I. H. (1994). Psychometric theory.

[B35-behavsci-16-00602] Oliver R. L. (1999). Whence consumer loyalty?. Journal of Marketing.

[B36-behavsci-16-00602] Podsakoff P. M., MacKenzie S. B., Lee J.-Y., Podsakoff N. P. (2003). Common method biases in behavioral research: A critical review of the literature and recommended remedies. Journal of Applied Psychology.

[B37-behavsci-16-00602] Ramos L. (2022). Marketing experiencial y elección de compra en servicios de barbería. Bachelor’s thesis.

[B38-behavsci-16-00602] Rather R. A. (2020). Customer experience and engagement in tourism destinations: The experiential marketing perspective. Journal of Travel & Tourism Marketing.

[B39-behavsci-16-00602] Sahyaja C., Harsha D., Sruthi G., Shankar C. (2026). Customer loyalty formation in the digital era: A mediated-moderated model of satisfaction, trust, brand image, engagement, and perceived value. World conference on information systems for business management.

[B40-behavsci-16-00602] Samson A., Voyer B. G. (2012). Two minds, three ways: Dual system and dual process models in consumer psychology. AMS Review.

[B41-behavsci-16-00602] Schmitt B. H. (1999). Experiential marketing: How to get customers to sense, feel, think, act, relate.

[B42-behavsci-16-00602] So K. K. F., King C., Sparks B. A., Wang Y. (2016). The role of customer engagement in building consumer loyalty to tourism brands. Journal of Travel Research.

[B43-behavsci-16-00602] Taipe-Abarca R., Vásquez M., Rodríguez J. (2025). Influence of digital marketing on customer loyalty in a digital training center in Trujillo. LACCEI International Multi-conference for Engineering, Education and Technology.

[B44-behavsci-16-00602] Vera-Martínez J. (2025). Service experiential attributes as the basis of customer value co-creation in technology product–services: A service-dominant logic perspective. Journal of Creating Value.

[B45-behavsci-16-00602] World Bank (2024). Peru macro poverty outlook (October 2024).

[B46-behavsci-16-00602] Yuan Y. H., Wu C. (2008). Relationships among experiential marketing, experiential value, and customer satisfaction. Journal of Hospitality and Tourism Research.

[B47-behavsci-16-00602] Zeithaml V. A. (1988). Consumer perceptions of price, quality, and value: A means-end model and synthesis of evidence. Journal of Marketing.

